# Silicon-based anti-herbivore defense in tropical tree seedlings

**DOI:** 10.3389/fpls.2023.1250868

**Published:** 2023-10-12

**Authors:** Marius Klotz, Jörg Schaller, Bettina M. J. Engelbrecht

**Affiliations:** ^1^ Leibniz Centre for Agricultural Landscape Research (ZALF), Müncheberg, Germany; ^2^ Deptartment of Plant Ecology, Bayreuth Center of Ecology and Environmental Research (BayCEER), University of Bayreuth, Bayreuth, Germany; ^3^ Smithsonian Tropical Research Institute (STRI), Balboa, Panama

**Keywords:** silica, plant defense, phytoliths, interspecific variation, herbivory

## Abstract

Silicon-based defenses deter insect herbivores in many cultivated and wild grass species. Furthermore, in some of these species, silicon (Si) uptake and defense can be induced by herbivory. Tropical trees also take up Si and leaf Si concentrations vary greatly across and within species. As herbivory is a major driver of seedling mortality and niche differentiation of tropical tree species, understanding anti-herbivore defenses is pivotal. Yet, whether silicon is a constitutive and inducible herbivory defense in tropical forest tree species remains unknown. We grew seedlings of eight tropical tree species in a full factorial experiment, including two levels of plant-available soil Si concentrations (-Si/+Si) and a simulated herbivory treatment (-H/+H). The simulated herbivory treatment was a combination of clipping and application of methyl jasmonate. We then carried out multiple-choice feeding trials, separately for each tree species, in which leaves of each treatment combination were offered to a generalist caterpillar (*Spodoptera frugiperda*). Leaf damage was assessed. Three species showed a significant decrease in leaf damage under high compared to low Si conditions (by up to 72%), consistent with our expectation of Si-based defenses acting in tropical tree species. In one species, leaf damage was increased by increasing soil Si and in four species, no effect of soil Si on leaf damage was observed. Opposite to our expectation of Si uptake and defense being inducible by herbivory damage, simulated herbivory increased leaf damage in two species. Furthermore, simulated herbivory reduced Si concentrations in one species. Our results showed that tropical tree seedlings can be better defended when growing in Si-rich compared to Si-poor soils, and that the effects of Si on plant defense vary strongly across species. Furthermore, Si-based defenses may not be inducible in tropical tree species. Overall, constitutive Si-based defense should be considered part of the vast array of anti-herbivore defenses of tropical tree species. Our finding that Si-based defenses are highly species-specific combined with the fact that herbivory is a major driver of mortality in tropical tree seedling, suggests that variation in soil Si concentrations may have pervasive consequences for regeneration and performance across tropical tree species.

## 1 Introduction

In tropical forests, most herbivory is caused by generalist leaf-chewing insects (Coley and Barone, 1996; Eichhorn et al., 2007; Bixenmann et al., 2016), leading to performance losses in the attacked plants (Zangerl et al., 2002). Specifically in seedlings, which are thought to be the most vulnerable life stage of tropical trees (Poorter, 2007), herbivory can reduce survival substantially (Eichhorn et al., 2010). Yet, anti-herbivore defenses can minimize the negative impacts of herbivory (Coley and Barone, 1996; Eichhorn et al., 2007). Many types of constitutive and inducible defenses that mechanically or chemically protect tropical plants from leaf damage have been identified (Coley and Barone, 1996; Eichhorn et al., 2007; Kursar et al., 2009; Barton, 2016). However, whether silicon-based defenses, which can effectively deter insect herbivores in many cultivated and wild grass species (Massey et al., 2006; Massey et al., 2007a; Singh et al., 2020), also act in tropical tree species has not been studied.

Plants take up silicon (Si) as dissolved mono-silicic acid from the soil, which is then carried with the transpiration stream to the leaves, where it accumulates as amorphous Si (Raven, 1983). Si accumulation is widespread among tropical trees and leaf Si concentrations vary strongly across species (Schaller et al., 2018). Leaf Si concentrations also vary within species (Schaller et al., 2018; Ishizawa et al., 2019), e.g. due to changes in plant-available soil Si concentrations (Klotz et al., 2023a), which differ considerably across tropical soils (Schaller et al., 2018; Ishizawa et al., 2019). Thus, the consequences of variation in soil and leaf Si concentrations for herbivory might be pervasive.

Si-based defenses act both physically and chemically (reviewed in Singh et al., 2020). Amorphous Si deposits make leaves more abrasive which can deter herbivores and thus reduces leaf damage (Massey et al., 2006; Massey et al., 2007a; Massey and Hartley, 2009; Hartley et al., 2015; Singh et al., 2020). Furthermore, ingestion of Si-rich diet can cause lasting damage to the insects’ mouth parts and digestive tract (Massey and Hartley, 2009), impairing their overall fitness (Massey et al., 2006; Massey and Hartley, 2009; Islam et al., 2020; Johnson et al., 2020; Islam et al., 2022). Besides such physical effects, Si can also improve plant defense by modulating molecular and biochemical plant responses to herbivory leading to greater production of defense-related enzymes and secondary metabolites (Fauteux et al., 2006; Ye et al., 2013; Hall et al., 2019; Singh et al., 2020). If constitutive Si-based defenses also act in tropical forest species, seedlings grown under high soil Si availability and/or having leaves with increased Si concentrations should show less damage by leaf-chewing insects.

There is strong evidence from grasses and crops that Si can act not only as a constitutive but also as an induced defense, i.e. herbivory damage can lead to greater Si uptake and accumulation in leaf tissues, which in turn reduces subsequent leaf damage (Massey et al., 2007a; McLarnon et al., 2017; Johnson et al., 2021). Leaf damage and signaling hormones involved in anti-herbivore responses, such as jasmonic acid and its derivative methyl jasmonate, have been shown to promote Si uptake and accumulation and might play a role in the induction of Si-based defenses (Ye et al., 2013; McLarnon et al., 2017; Hall et al., 2019; Johnson et al., 2021), although the exact interplay between Si and plant biochemistry is not yet fully understood (Hall et al., 2019). Thus, if Si-based defense is inducible in seedlings of tropical tree species, leaf damage should lead to increased leaf Si concentration and improved protection against herbivory.

Herbivory plays a central role in the ecology and evolution of tropical forest tree species, shaping their physiology and distribution (Coley and Barone, 1996; Fine et al., 2004; Kursar et al., 2009; Muehleisen et al., 2020), forest community composition (Fine et al., 2004; Kursar et al., 2009; Muehleisen et al., 2020) and other ecosystem processes, such as nutrient cycling (Metcalfe et al., 2014). Furthermore, projected shifts in rainfall and temperature with global climate change may influence plant-insect interactions, including herbivory (Hamann et al., 2021). Thus, understanding factors that influence herbivory rates in tropical forests is pivotal.

To test whether Si-based herbivory defenses act in seedlings of tropical tree species and whether they can be induced by herbivory, we experimentally exposed seedlings of eight common species to contrasting soil Si availability and simulated herbivory, and then carried out multiple-choice feeding trials with a generalist caterpillar. We hypothesized that within species (1) leaf damage should be lower in plants grown under high than low soil Si availability (and/or plants with high than low leaf Si concentrations), if Si acts as a herbivore defense. Additionally, (2) if Si accumulation is an inducible herbivore defense (a) leaf Si concentrations should increase, and (b) leaf damage should decrease after exposure to simulated herbivory.

## 2 Methods

We conducted a full-factorial experiment, including two levels of plant-available soil Si (-Si/+Si) and a simulated herbivory treatment (-H/+H), with potted seedlings of eight tropical tree species in Gamboa, Panama (9°070N, 79°420W). We then compared herbivore preferences for plants grown under the different treatment combinations in intraspecific multiple-choice feeding trials.

### 2.1 Study species and plant material

We studied eight tree species commonly found in tropical forests of central Panama and belonging to eight different families (**Table 1**). Species were selected based on the following criteria: (1) a wide range of leaf Si concentrations (K. Kitajima, J. Westbrook, and S. J. Wright, unpublished data) suggesting different physiological Si uptake capacities, (2) shade-tolerant species (Rüger et al., 2009), which make up the largest proportion of species in the area, and (3) availability of seeds before the onset of the experiment. In the following we refer to the species by their genus name or abbreviation (**Table 1**).

**Table 1:** Species included in the experiment with their scientific name, abbreviation, family, order, and the number of multiple-choice feeding trials conducted per species (N).

Scientific Name	Abbreviation	Family	Order	N
*Calophyllum longifolium* Willd.	CALOLO	Clusiaceae	Malpighiales	7
*Dendropanax arboreus* (L.) Decne. & Planch.	DENDAR	Araliaceae	Apiales	4
*Eugenia oerstediana* O.Berg	EUGEOE	Myrtaceae	Myrtales	5
*Herrania purpurea* (Pittier) R.E.Schult.	HERRPU	Malvaceae	Malvales	5
*Inga nobilis* Willd.	INGAQU	Fabaceae	Fabales	7
*Ormosia macrocalyx* Ducke	ORMOMA	Fabaceae	Fabales	5
*Randia armata* (Sw.) DC.	RANDAR	Rubiaceae	Gentianales	7
*Sorocea affinis* Hemsl.	SOROAF	Moraceae	Rosales	6

Seeds were collected in forests of the Panama Canal area in October and November 2019 from at least three individual trees per species with a minimum distance of 100 m between them. Seeds were germinated and raised until cotyledon stage or development of first foliage leaves in trays on a nutrient- and Si-poor substrate consisting of 50% local forest soil and 50% washed river sand (equivalent to substrate for Si treatment, see below).

Seedlings were then transplanted into individual pots (Deepot Cells, Stuewe & Sons, Oregon, USA; diameter: 6.5 cm, depth: 36 cm) with the two experimental substrates (+Si or -Si, see below) in December 2019 or January 2020. The mean experimental growing period varied between ca. 11 - 12.5 months, depending on the species. The species’ mean durations of the growing period did not correlate (Pearson correlation) with their leaf Si concentrations of the +Si/-H treatment. Throughout the experiment, all plants were kept well-watered, under intermediate light conditions (ca. 10% of full sunlight) and protected from rainfall. Additionally, -H plants were protected from herbivores by fly screens. The position of species and treatments was randomized. We fertilized each plant using 20 ml of a half strength Hoagland solution three times within the first three months of the experiment to ensure survival of species associated to nutrient-rich soils.

### 2.2 Si treatment

We manipulated plant-available soil Si concentrations (also termed soil Si in the following). Plants of the -Si treatment grew in a Si- and nutrient-poor substrate consisting of 50% local forest soil (Cerro Pelado, selected based on Schaller et al., 2018 and Condit et al., 2013) and 50% washed river sand. Plants of the +Si treatment grew in the same substrate supplemented with amorphous Si (Aerosil 300, Evonik Industries AG, Essen, Germany; 18 g L^-1^ substrate), a hydrophilic pyrogenic silicon dioxide. Aerosil 300 has similar chemical and physical properties as biogenic amorphous Si (Schaller et al., 2020) and supplementing soils with Aerosil 300 increases plant-available Si without changing soil pH (J. Schaller, unpublished data). The amorphous Si and substrate were mixed thoroughly. Resulting plant-available soil Si concentrations were 5.23 and 18.27 mg kg^-1^ for the -Si and +Si treatment, respectively (for analyses see below), corresponding to the minimum and mean values found in the region (Schaller et al., 2018).

### 2.3 Simulation of herbivory

To elicit potential inducible herbivory defenses we treated half of the plants of both Si treatments with simulated herbivory (+H), a combination of clipping and application of methyl jasmonate (MeJa), a hormone inducing systemic defense responses (Mithöfer and Boland, 2012). First, two leaves of each +H plant were clipped along the lamina edges with scissors removing about 50% of the leaf area. Then MeJa mixed with lanolin (4.5 µmol in 100 mg lanolin paste) was applied to the surface of the leaves (10 mg on an area of ca. 0.5 x 0.5 cm per leaf). The -H plants were not clipped and received the same amount of lanolin paste without MeJa. The treatment was repeated six times throughout the whole experimental period, with the last application not more than three weeks before the onset of the feeding trials. Leaf clipping combined with MeJa application enabled us to standardize the intensity and amount of (simulated) herbivory across and within species, although some additional chemical and physical stimuli of natural herbivores triggering plant responses may be missing (Waterman et al., 2019).

### 2.4 Generalist herbivore

Multiple-choice feeding trials were conducted with 3rd or 4th instar caterpillars of the moth *Spodoptera frugiperda* (J.E. Smith), a generalist herbivore that does not occur in forest habitats in Panama. Using a herbivore that does not co-occur with the focal plant species enabled us to avoid potential confounding effects of co-evolution, and using a generalist allowed us to use the same herbivore across tree species. The caterpillars were picked from corncobs purchased at a local marked the day before the respective feeding trials, were starved for 10h and kept in the laboratory under standardized conditions. Each individual caterpillar was only used for one feeding trial (see below) to prevent learning effects.

### 2.5 Feeding trials

We carried out multiple-choice feeding trials separately for each tree species. In each trial, we offered one leave disk per treatment combination to one caterpillar in a Petri dish (90 mm diameter) and assessed leaf damage. Additionally, a leaf disk from a standard plant species (*Ixora coccinea*) was included in each trial, but was not considered further in the analyses. Four to seven trials were conducted per species (N = 4–7, **Table 1**). One seedling per treatment combination was randomly selected for each trial. Fully developed leaves or leaves of the same developmental stage were selected, cleaned, stored in plastic bags and kept in a fridge. Directly before the trials leaf disks of 2 cm² size were punched out with a cork borer (avoiding the main leaf rib). They were pinned to moist sponges to retain tissue moisture and then placed into a Petri dish. The leaf disks were arranged in a circle with the caterpillar placed in the center. The positions of the four treatment groups was randomized and recorded to identify them after the trial. Each trial took 6h. If the caterpillar did not feed on any of the leaf disks the trial was repeated with a new caterpillar and leaf disks from new leaves. In cases where the caterpillar did not feed again, a second (and sometimes third) repetition was carried out, exchanging only the caterpillar (not the leaves, to ensure to keep enough leaf material for Si analysis, see below) and extending the feeding period to 8h.

After the feeding trials, the remaining area of the leaf disks (LA_remain_) was photographed (NIKON Coolpix 500) and measured using image software ‘ImageJ’ (Schneider et al., 2012). For the statistical analysis, we calculated the proportion of leaf area consumed as 1-(LA_remain_/LA_offered_). In the figures we present the percentage of leaf area consumed.

Some individuals of one species (*Dendropanax*) showed signs of herbivory damage before the feeding trials in the -H treatment. We kept data of these individuals in the analyses, so that the effect of simulated herbivory on subsequent leaf damage should be interpreted with care for this species.

### 2.6 Si analysis of leaves and soil

The leaves that remained on the plants (i.e. leaves not used in the feeding trials plus the leaf area remaining after punching out the leaf disks) were harvested and cleaned to remove any potential residual soil material. They were oven-dried for 48h at 65°C and ground to a fine powder. Leaf Si was extracted for 5 h by an alkaline method using 30 mg of leaf material and 30 ml of 0.1 M sodium carbonate solution (Na_2_CO_3_) in a regularly shaken water bath at 85 C° (Schaller et al., 2018). The solution was centrifuged (3000×g, for 5 min) and passed through a 0.45 µm cellulose acetate filter (Rausch, 2021).

Both experimental soil substrates were sampled before the start of the experiment to analyze the maximum plant-available soil Si concentration the plants were exposed to (one sample per Si level). The samples were air-dried, crushed to break up large aggregates, and sieved to remove roots and stones. Plant-available Si was extracted in CaCl_2_ following Schaller et al. (2018). Three g of sifted soil were shaken with 30 ml of 0.01 M CaCl_2_ for 1 h at ambient laboratory temperature. The suspension was centrifuged (8000×g, for 10 min) and the supernatant decanted and subsequently passed through a 0.2 μm syringe filter.

The Si concentration of the leaf and soil extracts was determined with inductively coupled plasma optical-emission spectrometry (ICP-OES) using a Varian Vista-Pro Radial element analyzer (Varian Inc., Palo Alto, USA).

### 2.7 Statistical analyses

#### 2.7.1 Effect of soil Si and simulated herbivory on leaf damage

To assess the effect of the soil Si and simulated herbivory treatments as well as their interaction on leaf damage we run zero-inflated generalized linear mixed-effect models (ZIGLMM) for each species, using the function ‘glmmTMB’ from the R-package ‘glmmTMB’ (Brooks et al., 2017).As our data was proportional and contained many zeros we assumed the residuals to be beta-distributed and zero-inflated (Geissinger et al., 2022). If the whole leaf disk was consumed, i.e. if leaf damage = 1, we subtracted a trace value of 0.0001, as responses = 1 cannot be modeled with the R-function we used. We included “Feeding trial” as random effect. For two species the full model did not converge so we re-run the model without the interaction term (*Ormosia*) or without the random effect (*Dendropanax*). We assessed significance of the factors using 90% and 95% confidence intervals from parametric bootstrapping (based on 10000 iterations in which the model successfully converged). We predicted marginal means for all four factor levels based on the ZIGLMMs using the R-package ‘ggeffects’ (Lüdecke, 2018), yet without consideration of the zero-inflation component. We assessed the effect size of soil Si and simulated herbivory on leaf damage as the % increase or decrease of the marginal means of the +Si and +H compared to control (i.e. the -Si and -H treatments), respectively. We performed model diagnostics using the R-package ‘DHARMa’ (Hartig, 2022).

#### 2.7.2 Effect of soil Si and simulated herbivory on leaf Si concentration

To test whether the soil Si and the (simulated) herbivory treatments and their interaction had an effect on leaf Si concentrations we run ANOVA for each species. We applied graphical model diagnostics to ensure normality and homogeneity of residuals. To interpret the effects of soil Si and simulated herbivory, we predicted marginal means for all factor levels based on the ANOVAs.

#### 2.7.3 Effect of leaf Si concentrations on leaf damage

To assess the direct effect of leaf Si concentrations on leaf damage for each species we also run separate ZIGLMMs (see above) for each species, including “Feeding trial” as random effect (except for *Dendropanax*). We assessed the significance of the slope estimate using 90% and 95% confidence intervals from parametric bootstrapping (based on 10000 iterations in which the model successfully converged). We assessed the effects of leaf Si concentrations on leaf damage for each species as the difference between the predicted marginal means of leaf damage at the lowest and highest leaf Si concentration measured in each species, yet without consideration of the zero-inflation component.

We considered *p*-values of *p* < 0.05 as evidence for a significant effect, while 0.05 < *p* < 0.1 indicated weak evidence, and *p* > 0.1 no evidence for an effect (compare Muff et al., 2022). All statistical analyses were performed in R version 4.2.1 (R Core Team, 2022).

## 3 Results

### 3.1 Effects of soil Si and simulated herbivory on leaf damage

**Figure 1:**
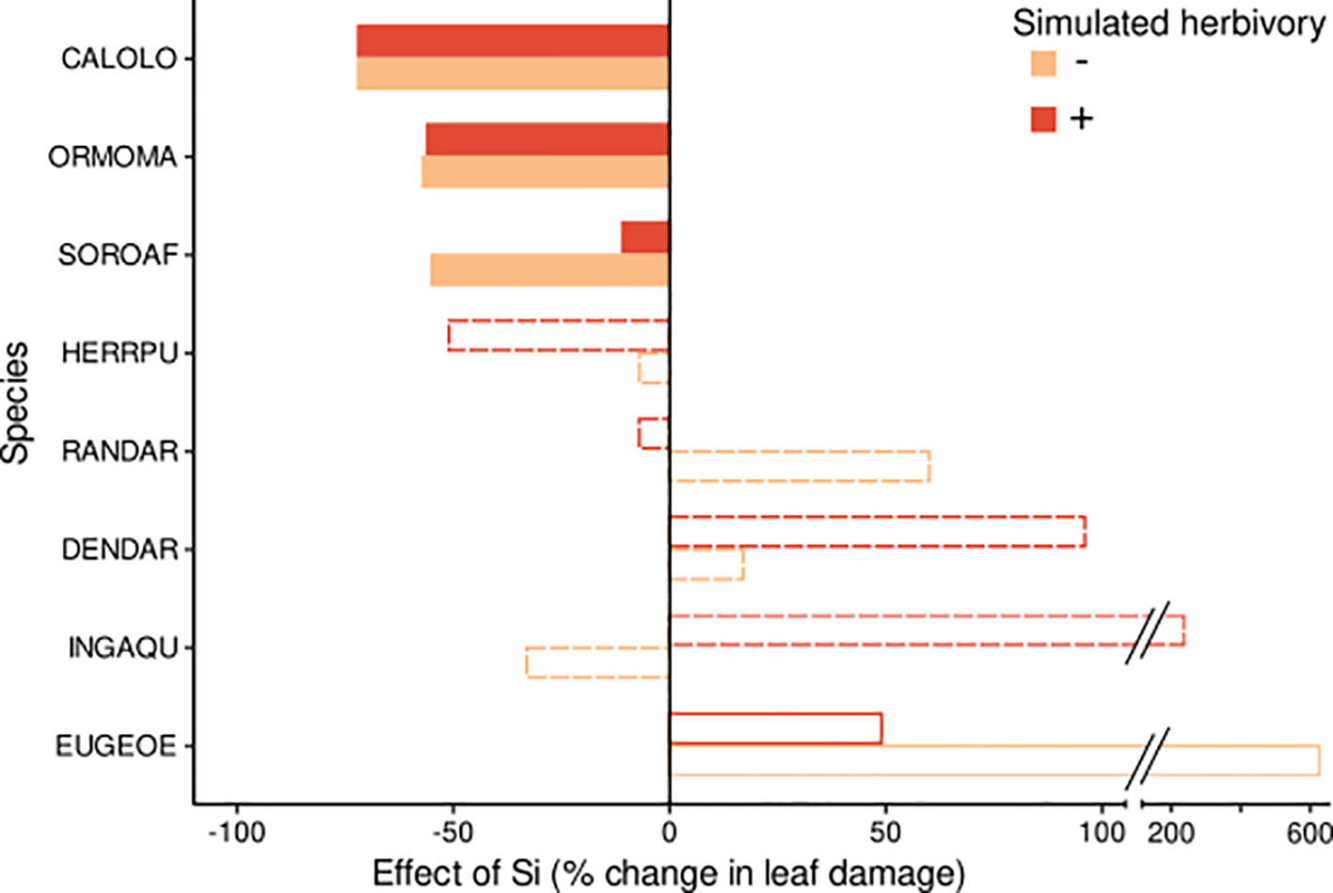
Effect size of the Si treatment on leaf damage for eight tropical tree species. Effects are shown for plants without herbivory (orange bars) and for plants subjected to simulated herbivory (red bars). The effect size indicates the % change of leaf damage in the predicted marginal means in the +Si relative to the -Si treatment. Significant effects and effects with weak or no evidence (for details see **Table 2**) are indicated by filled, empty and dotted bars, respectively. Species codes are given in **Table 1**.

**Figure 2:**
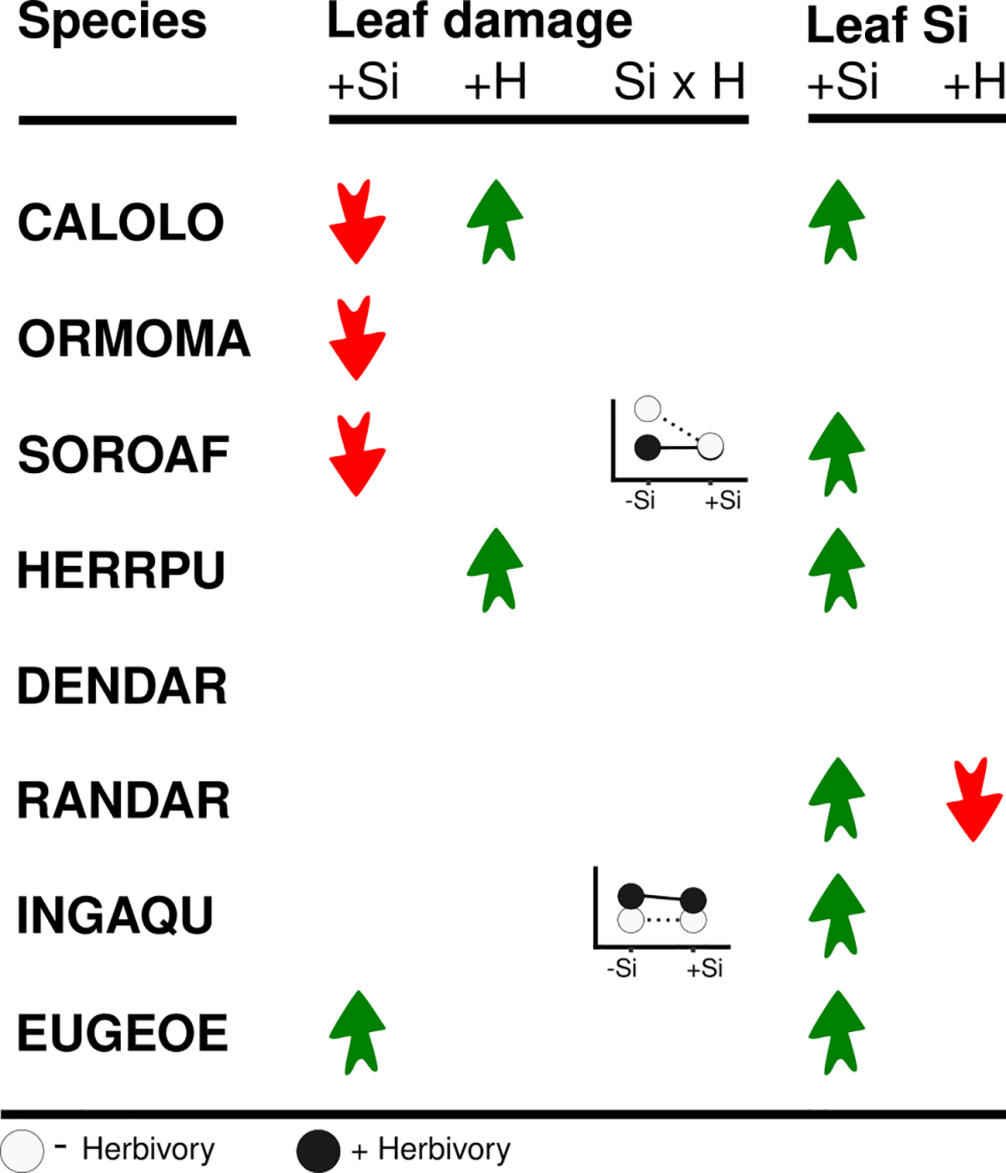
Overview of the effects of high soil Si (+Si) and simulated herbivory (+H) on leaf damage and Si concentrations. Red and green arrows denote significant reductions and increases, respectively. Graphs show significant interactions between soil Si and simulated herbivory (Si x H).

Several species showed significant effects of Si and/or simulated herbivory on leaf damage, but the size and direction of responses was not consistent across species. Three species (*Calophyllum*, *Ormosia* and *Sorocea*) showed a significant decrease in leaf damage under high compared to low Si conditions (by 11% to 72%, **Figures 1**, **2**; **Table 2**; **Figure S2**), consistent with the hypothesis of Si-based herbivory defenses. In contrast, in one species (*Eugenia*), we found weak evidence for an increase in leaf damage under high compared to low Si conditions. In the four remaining species no evidence for an effect of soil Si on leaf damage emerged (*Herrania, Dendropanax, Randia, Inga*).

**Table 2:** Effect of plant-available soil Si (Si), simulated herbivory (H) and their interaction (Si:H) on leaf damage (% of leaf area removed) in intraspecific multiple-choice feeding trials with seedlings of eight tropical tree species.

	+Si			+H			Si x H		
	β	CI95%	CI90%	β	CI95%	CI90%	β	CI95%	CI90%
**CALOLO**	**-1.36**	-2.64 - -0.32	-2.38 - -0.52	*0.88*	-0.02 - 1.80	0.18 - 1.60	-0.12	-1.56 - 1.43	-1.29 - 1.12
**ORMOMA**	**-0.90**	-1.72 - -0.22	-1.58 - -0.34	0.08	-0.64 - 0.81	-0.50 - 0.67	†		
**SOROAF**	**-1.02**	-1.6 - -0.45	-1.48 - -0.57	-0.35	-0.90 - 0.19	-0.77 - 0.08	**0.87**	0.02 - 1.68	0.22 - 1.51
**HERRPU**	-0.08	-2.48 - 1.04	-1.24 - 0.73	**7.27**	3.84 - 10.01	5.26 - 9.41	-5.29	-8.04 - 0.00	-7.46 - 0.00
**DENDAR**	0.18	-0.96 - 1.35	-0.73 - 1.14	-0.54	-1.90 - 0.70	-1.67 - 0.48	0.57	-1.06 - 2.28	-0.76 - 2.00
**RANDAR**	0.54	-0.63 - 1.77	-0.41 - 1.57	0.56	-0.56 - 1.77	-0.35 - 1.57	-0.62	-2.29 - 0.93	-2.00 - 0.65
**INGAQU**	-0.44	-1.32 - 0.51	-1.16 - 0.28	0.19	-0.56 - 1.10	-0.43 - 0.91	**2.29**	0.74 - 3.42	1.14 - 3.22
**EUGEOE**	*2.23*	0.00 - 2.75	0.13 - 2.67	1.39	-0.34 - 1.94	-0.20 - 1.85	-1.76	-2.36 - 0.38	-2.24 - 0.27

Slope estimates (β) of zero-inflated generalized linear mixed-effect models and 95% (CI95%) and 90% (CI90%) bootstrapped confidence intervals are shown. Significant effects (i.e. the 95% CI does not include zero) and effects with weak evidence (i.e. only the 90% CI does not include zero) are marked bold and italic, respectively. Species codes are given in **Table 1**.

^†^ Convergence error in model with interaction term.

**Figure 3:**
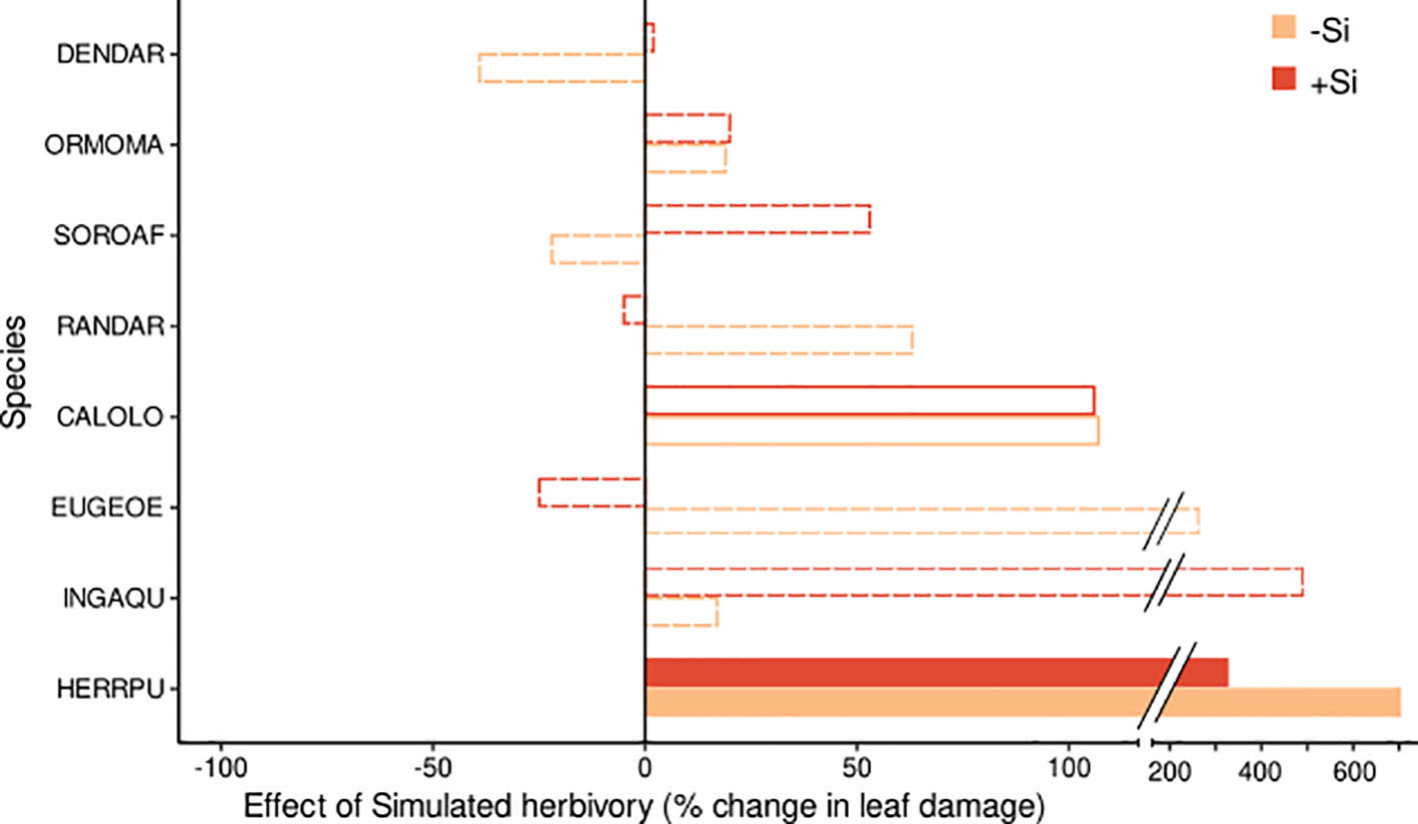
Effect size of the simulated herbivory treatment on leaf damage for eight tropical tree species. Effects are shown for plants of the +Si (red bars) and -Si (orange bars) treatment. The effect size indicates the % change of leaf damage in the predicted marginal means in the +H relative to the -H treatment. Significant effects and effects with weak or no evidence (for details see **Table 2**) are indicated by filled, empty and dotted bars, respectively. Species codes are given in **Table 1**.

Simulated herbivory significantly increased leaf damage (by up to 700%, **Figures 2**, **3**; **Table 2**) in one species (*Herrania*), and we found weak evidence for an positive effect in another species (*Calophyllum*). The remaining six species showed no evidence for an effect of simulated herbivory on leaf damage.

Significant interactive effects of soil Si and simulated herbivory on leaf damage emerged in two species (**Table 2**), yet they were not in line with our hypothesis of induced Si-based defense (i.e. lowest herbivory in +Si plants receiving simulated herbivory). Instead, the decrease of leaf damage with soil Si was stronger in plants without simulated herbivory in one species (*Sorocea*). In another species (*Inga*) Si fertilization increased leaf damage in plants treated with simulated herbivory and decreased it in plants without simulated herbivory.

### 3.2 Effects of soil Si and simulated herbivory on leaf Si concentrations

**Figure 4:**
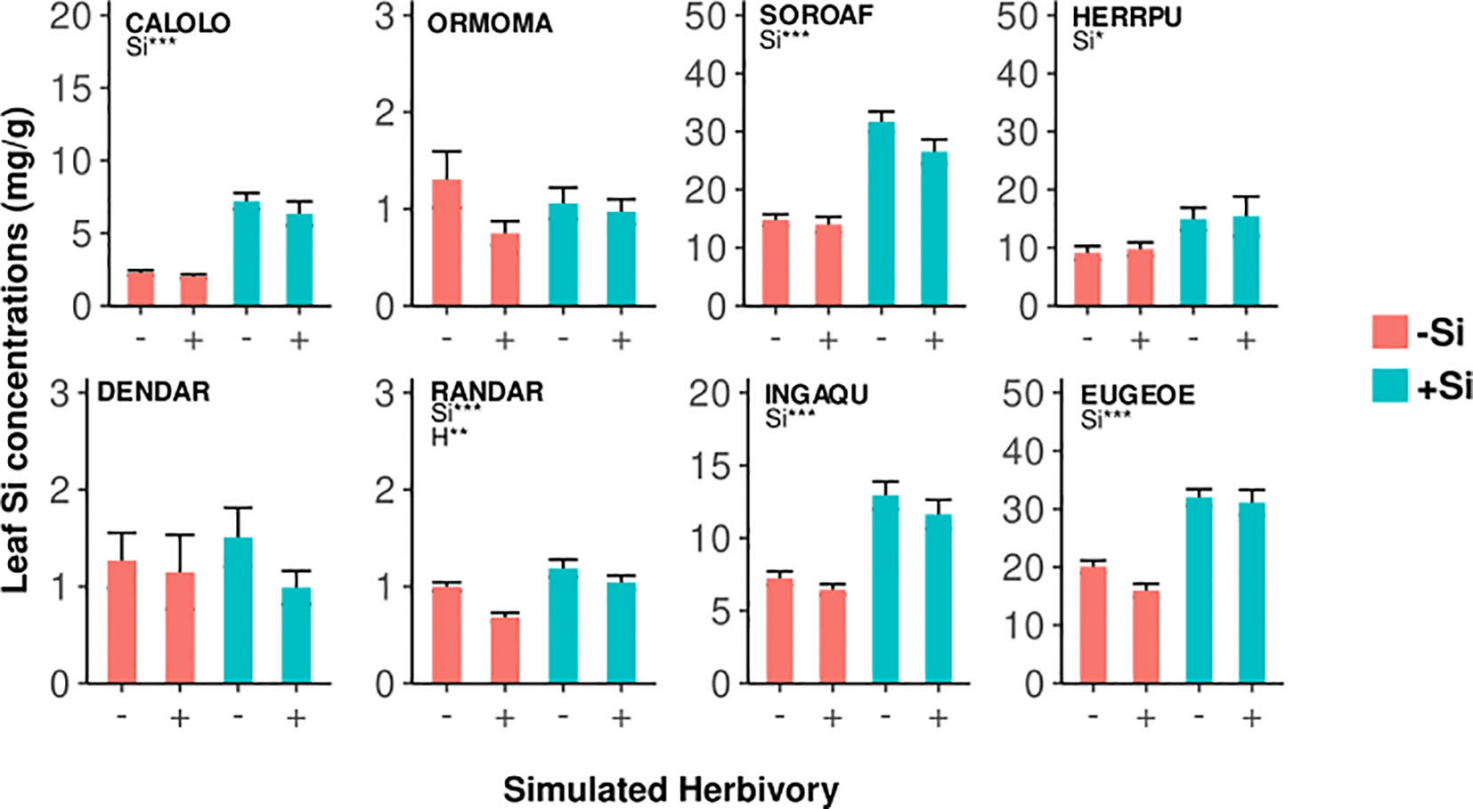
Leaf Si concentrations in seedlings of eight tropical tree species under different plant-available soil Si (-Si/+Si) and simulated herbivory (-H/+H). Results of ANOVA are shown (significant effects of Si (Si), simulated herbivory (H), and the interaction (H:Si); *p < 0.05, **p < 0.01, ***p < 0.001). Values are means ± SD per treatment combination and species. Species codes are given in **Table 1**.

In six of the eight species leaf Si concentrations were significantly higher in plants growing under high Si compared to low Si conditions (up to 219%, **Figures 2**, **4**; **Table 3**). In two species (*Dendropanax, Ormosia*), which showed the lowest Si uptake capacity out of all species we studied (**Figure S1**), leaf Si concentrations did not increase with higher soil Si. Overall, the species showed a 30-fold variation in Si uptake capacity (i.e. the leaf Si concentrations of +Si plants not treated with simulated herbivory).

**Table 3:** Effect of plant-available soil Si (Si), simulated herbivory (H) and their interactions (H:Si) on leaf Si concentrations in seedlings of eight tropical tree species.

	H			Si			H:Si		
	df_nom, den_	F	*p*	df_nom, den_	F	*p*	df_nom, den_	F	*p*
**ORMOMA**	1, 15	2.47	0.137	1, 15	0.02	0.899	1, 15	1.40	0.254
**CALOLO**	1, 24	1.17	0.290	1, 24	82.51	**<0.001**	1, 24	0.36	0.557
**DENDAR**	1, 12	1.16	0.304	1, 12	0.02	0.895	1, 12	0.46	0.512
**EUGEOE**	1, 16	2.63	0.124	1, 16	78.89	**<0.001**	1, 16	1.13	0.304
**SOROAF**	1, 20	3.42	0.079	1, 20	84.65	**<0.001**	1, 20	1.88	0.186
**INGAQU**	1, 24	2.92	0.100	1, 24	31.92	**<0.001**	1, 24	1.02	0.322
**RANDAR**	1, 24	12.18	**0.002**	1, 24	17.25	**<0.001**	1, 24	1.60	0.219
**HERRPU**	1, 16	0.07	0.794	1, 16	7.32	**0.016**	1, 16	0.00	0.965

Results of ANOVAs are shown. Significant responses (p < 0.05) are shown in bold. Species codes are given in **Table 1**.

Simulated herbivory did not lead to an increase of leaf Si concentrations in any of the species, again contrary to our expectation of Si being an induced defense. In contrast, simulated herbivory reduced leaf Si concentrations in one species (*Randia*), by about 32% and 12% under low and high Si availability, respectively.

### 3.3 Effect of leaf Si concentration on leaf damage

**Figure 5:**
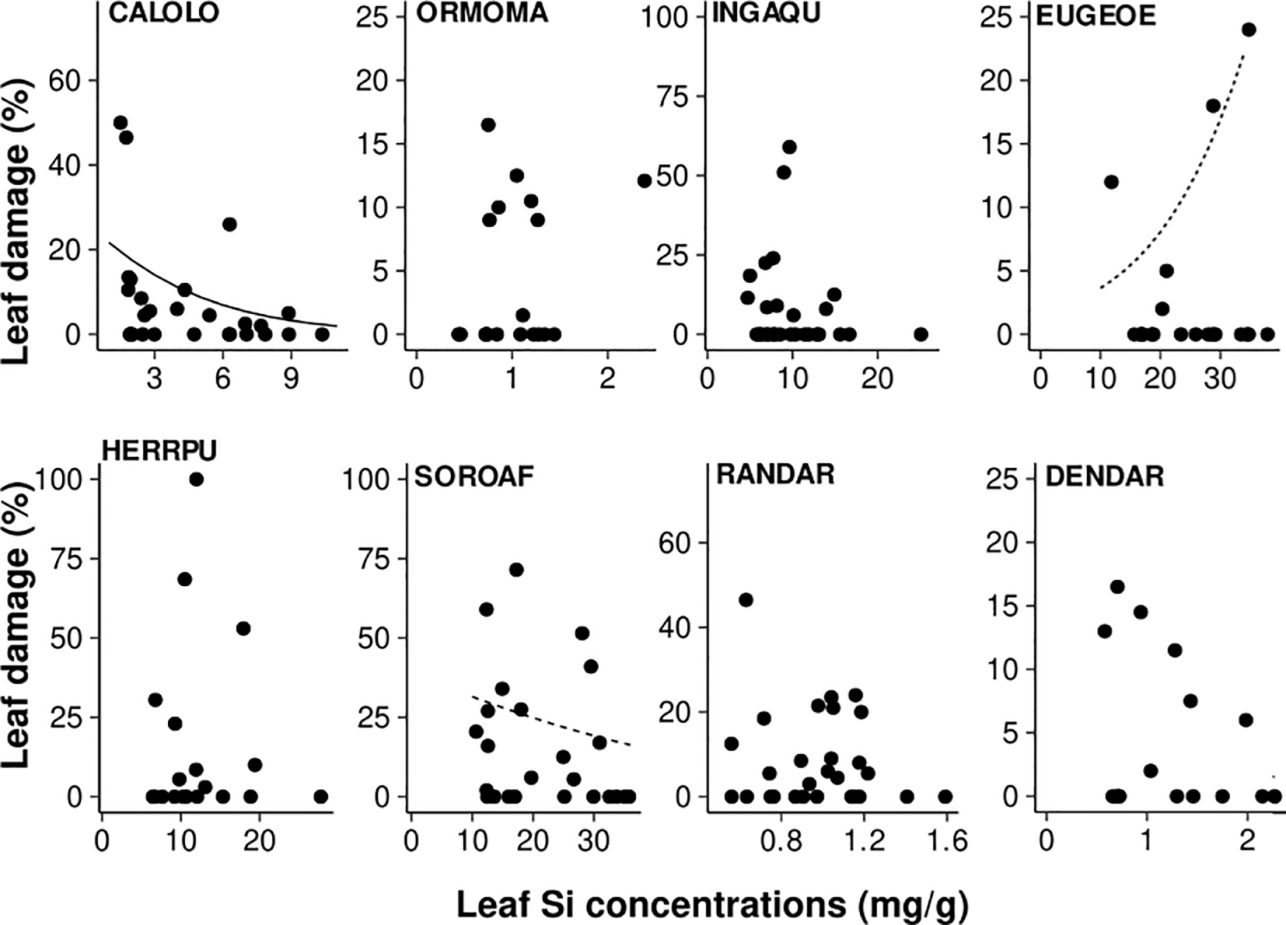
Effect of leaf Si concentrations on leaf damage (% leaf area removed). Results of zero-inflated generalized linear mixed-effect models are shown (bold line: *p* < 0.05, dashed line: *p* < 0.1; significance was assessed based on bootstrapped confidence intervals). Species codes are given in **Table 1**).

Leaf Si concentrations affected leaf damage, both negatively and positively, in three of the eight species studied (**Figure 5**; **Table 4**), while leaf Si concentrations did not influence leaf damage in the remaining five species. Increasing leaf Si concentrations significantly decreased leaf damage in one species (by 91%, *Calophyllum*), in line with our hypothesis, and, in another species (*Sorocea*), we found weak evidence for such an effect. By contrast, in one species (*Eugenia*) there was weak evidence for the opposite effect, i.e. leaf damage increased with increasing leaf Si concentration (by 685%).

**Table 4:** Effect of leaf Si concentrations on leaf damage (% leaf area removed) in intraspecific multiple-choice feeding trials.

	Foliar Si concentration		
	β	CI95%	CI90%
**CALOLO**	**-0.26**	-0.46 - -0.10	-0.42 - -0.13
**ORMOMA**	0.08	-1.49 - 1.52	-1.12 - 1.20
**SOROAF**	*-0.03*	-0.07 - 0.00	-0.06 - -0.01
**HERRPU**	0.01	-0.42 - 0.12	-0.34 - 0.06
**DENDAR**	-0.31	-0.97 - 0.39	-0.71 - 0.14
**RANDAR**	-0.86	-2.64 - 0.8	-2.32 - 0.52
**INGAQU**	-0.02	-0.18 - 0.17	-0.14 - 0.13
**EUGEOE**	*0.08*	-0.02 - 0.19	0.01 - 0.17

Slope estimates (β) of zero-inflated generalized linear mixed-effect models and 95% (CI95%) and 90% (CI90%) bootstrapped confidence intervals are shown. Significant effects (i.e. the 95% CI does not include zero) and effects with weak evidence (i.e. only the 90% CI does not include zero) are marked bold and italic, respectively. Species codes are given in **Table 1**.

## 4 Discussion

Effects of soil or leaf Si concentrations and simulated herbivory on leaf damage emerged in seedlings of several tropical tree species and exhibited strong interspecific variation. Higher soil and/or leaf Si concentrations decreased leaf damage by a generalist herbivore in three of the eight species we studied, indicating that Si improved their herbivory defense. In the other species, however, leaf damage either showed no or even a positive relationship to soil Si and leaf Si concentrations. We did not find evidence for inducible Si-based defenses.

### 4.1 Si-based defenses in tropical seedlings

In about 40% of the species we studied Si-based defenses reduced leaf damage, consistent with our hypothesis. Furthermore, the species in which Si improved defense varied greatly in their Si uptake capacities (up to 30-fold). This is in line with work on several grass and crop species, including low-accumulating dicots, that showed reduced leaf damage by insect herbivores in plants fertilized with Si (e.g. Massey et al., 2006; Ryalls et al., 2017; Islam et al., 2020). The range of reduction of leaf damage with Si fertilization in our study, i.e. a 11% to 72% reduction, is similar to Massey et al. (2006), who reported a ca. 43% to 75% reduction across five grass species. In contrast to our finding of Si-based defenses occurring only in a subset of tropical tree species, effects of Si consistently emerged in all grass species studied (Massey et al., 2006), probably because Si-based defenses are one of the main defense types in grasses (Massey et al., 2007a; Huitu et al., 2014). Tropical trees, however, have evolved a wide diversity of chemical as well as physical herbivore defenses, which vary substantially across species (Coley and Barone, 1996; Kursar et al., 2009; Eichhorn et al., 2010; Barton, 2016). Hence, finding evidence for Si-based defenses in about 40% of the tree species indicates that Si can play an important but so far widely ignored role in anti-herbivore defense in tropical forests. Conversely and opposite to our expectations, high leaf Si concentrations led to increased leaf damage in one species (Eugenia), indicating that, in some species, Si-rich leaves can also be more susceptible to herbivory. This might be due to increases in nutritional quality, e.g. tissue N and/or P concentrations, with higher leaf and/or soil Si concentrations as previously shown for tropical tree and crop species (e.g. Neu et al., 2017, Klotz et al., 2023a). To our knowledge we are the first to show that Si-based defenses act in seedlings of tropical tree species. Our results suggest that Si should be considered part of the vast array of anti-herbivores defenses in tropical trees (Coley and Barone, 1996; Kursar et al., 2009; Barton, 2016).

The mechanisms underlying the Si-based defenses we observed likely varied across our study species. Si-based defenses were independent of the species’ Si uptake capacity, i.e. they can occur in both high- and low-accumulating species. In two species (*Calophyllum* and *Sorocea*) higher leaf Si concentrations were directly related to reduced leaf damage. In these species, amorphous Si deposits may have directly lead to more abrasive leaves, as found elsewhere (Massey et al., 2006; Massey and Hartley, 2009). Indeed, the best known direct constitutive anti-herbivore effect of Si is a higher abrasiveness of Si-rich leaves, which can wear down the herbivores’ mouthparts and reduce their digestive efficacy (Massey et al., 2006; Massey and Hartley, 2009; Hartley et al., 2015; Singh et al., 2020). In another of our study species (*Ormosia*), however, the reduction in leaf damage was not related to higher leaf Si concentrations but only soil Si had an effect on leaf damage, suggesting that the Si-enriched soil conditions must have indirectly influenced defensive leaf properties. Such indirect effects may include Si-mediated changes in soil nutrient availability and plant nutrient status (see above), which may also influence the production and composition of defensive secondary metabolites (Moore et al., 2014), and lead to changes in nutritional quality. Alternatively, high soil Si may have modulated the morphology and/or location of Si deposits in leaves in a way that improved their defensive properties (Hartley et al., 2015; Schaller et al., 2022). Indeed, previous work on grass species suggested that the morphology and location of leaf Si deposits might be more important factors for Si’s protective effects than leaf Si concentrations per se (Hartley et al., 2015). Changes in the morphology and location of Si deposits without concurrent changes in leaf Si concentrations might be related to differences in the amount and/or structure of silicification templates, such as cell walls (Kumar et al., 2017). Yet, whether and how leaf Si deposits of *Ormosia* have changed was not assessed in our study. Overall, the mechanisms underlying Si-based defenses, e.g. whether or not high soil Si alone is sufficient to improve herbivory defense, vary across species and disentangling this variation will contribute to our ecological understanding of Si in tropical forests.

Despite the large effect sizes and robust calculation of confidence intervals (see above), our sample size of n = 4-7 is relatively small and the minimum for feeding trials, which remains a caveat of our study.

### 4.2 Si-based defenses were not inducible

In none of our species simulated herbivory led to higher leaf Si concentrations or to lower leaf damage under high soil Si, suggesting that Si uptake and the associated improvements in herbivory defense may not be inducible in tropical tree seedlings. Previous work had demonstrated that Si uptake can be induced by natural and simulated herbivory in grasses and some dicot species (Massey et al., 2007b; Quigley and Anderson, 2014; McLarnon et al., 2017; Islam et al., 2020; Johnson et al., 2020). Application of MeJa, which was also a part of the simulated herbivory treatment in our study, has successfully induced Si uptake in several previous studies (e.g. Hall et al., 2020; Johnson et al., 2021). An ecological explanation for not finding induced Si-based defenses in the tropical tree species may be that under conditions of constantly high herbivore pressure, such as in tropical forests, induced herbivory defense is overall less cost-effective and thus less common than constitutive defense (but see Barton, 2016; Bixenmann et al., 2016). In fact, the costs of Si uptake and thus Si-based defenses can be high (Garbuzov et al., 2011; de Tombeur et al., 2023), so that additional herbivory-induced Si uptake might not be worthwhile, e.g. because it may not add to the protection already present in the form of constitutive Si-based defense. We note though that in some studies repeated leaf damage was necessary to induce Si uptake (Massey et al., 2007b; Hartley and DeGabriel, 2016), and that simulated herbivory through leaf clipping and MeJa application may be less effective in inducing Si uptake than natural herbivory, potentially due to the absence of chemical and physical stimuli associated with herbivore damage, e.g. saliva, that trigger additional (hormonal) plant responses (Hartley and DeGabriel, 2016; Waterman et al., 2019). Thus, the amount of MeJa applied and/or the amount or mode of mechanical damage applied in our study might not have been adequate to induce Si uptake. Si accumulation may also have occurred only locally in damaged leaves (but see Islam et al., 2020, see Thorne et al., 2023) and thus remained undetected in our study since we pooled the total leaf biomass per individual to measure Si concentrations. At this point we can therefore not conclusively rule out that induced Si-based defenses occur in some tropical tree species.

## 5 Ecological implications

Considering the vast spatial variation of plant-available soil Si in tropical forests (Schaller et al., 2018; Ishizawa et al., 2019) Si-based defenses and their strong intra- and interspecific variation might have pervasive implications for the ecology of tropical tree seedlings. Firstly, defensive effects of both soil and leaf Si have been suggested to provide an alternative to C-based defense compounds (Raven, 1983; Schaller et al., 2012). This may be especially important in seedlings in the shaded forest understory of tropical forests, which are strongly light- and thus C-limited (Chazdon, 1988). Secondly, for species in which Si has protective effects, seedlings growing in sites with Si-rich soil should be better protected against herbivores than conspecifics on Si-poor soil, contributing to intraspecific performance variation. Thirdly, the pronounced interspecific differences of the efficacy of Si-based defenses may lead to changes in performance rankings and competitive balance across sites varying in plant-available soil Si (Garbuzov et al., 2011), with repercussions for tree community composition. Furthermore, the ecology of herbivores may also be affected by variation of soil Si, because a Si-rich diet not only reduces the herbivores’ fitness, but it can also both increase and decrease their susceptibility to predation and parasitism (Ryalls et al., 2017; Hall et al., 2021; Islam et al., 2022). Overall, our results suggest Si-based defenses in tropical tree seedlings may have pervasive consequences for seedling performance and ecological processes, such as plant-herbivore interactions, and thus should be considered more in further studies.

## Data availability statement

The datasets presented in this study can be found in online repositories. The names of the repository/repositories and accession number(s) can be found below: Dryad Digital Repository at https://doi.org/10.5061/dryad.j0zpc86mf (Klotz et al., 2023b).

## Author contributions

BE and JS conceived the idea. BE, JS and MK designed the study. The implementation of the experiment was coordinated by MK. Chemical analyses were coordinated by BE, JS and MK. The data were analyzed, and figures and tables created by MK with input from BE and JS. MK wrote the manuscript. All co-authors revised and commented subsequent drafts and gave final approval for publication.
