# Supplementary material for: Silicon-based anti-herbivore defense in tropical tree seedlings
Source: Front Plant Sci. 2023 Oct 12;14:1250868. doi: 10.3389/fpls.2023.1250868 (PMC10602810; doi:10.3389/fpls.2023.1250868)
Supplement: Supplementary file 1 [file DataSheet_1.docx]

Supplementary Material

Silicon-based anti-herbivore defense in tropical tree seedlings

Marius Klotz^*^, Jörg Schaller, Bettina M. J. Engelbrecht

*** Correspondence:**Corresponding Author
marius.klotz@uni-bayreuth.de

## Supplementary Figures


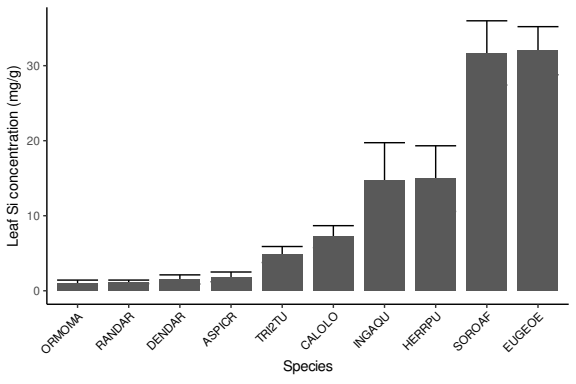


**Supplementary Figure 1.** The species’ Si concentrations under Si+ / H- treatment, reflecting their Si uptake capacities.


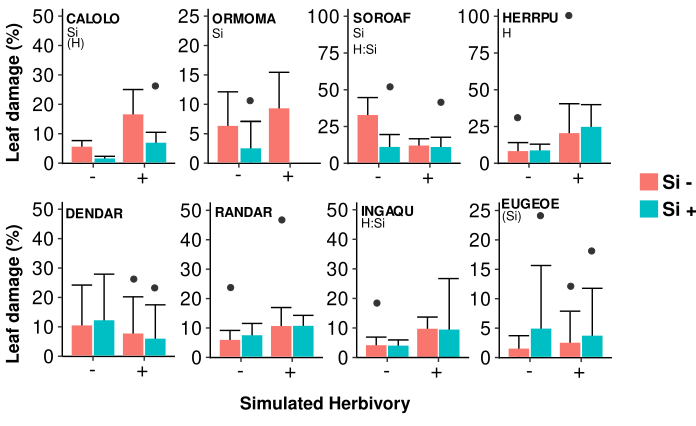
**Supplementary Figure 2.** Leaf damage (% of leaf area removed) under different soil Si (Si+, Si-) and simulated herbivory (H+, H-) in intraspecific multiple-choice feeding trials with seedlings of eight tropical tree species. Values are means ± SD and outliers per treatment combination and species. Results of zero-inflated generalized linear mixed-effect models are shown (significant effect of Si (Si), simulated herbivory (H) and interaction (H:Si). Significant effects (i.e. the 95% CI does not include zero) and effects with weak evidence (i.e. only the 90% CI does not include zero) are shown with and without parentheses, respectively. Species codes are given in Table 1.
